# Short-term exposure to air pollution (PM_2.5_) induces hypothalamic inflammation, and long-term leads to leptin resistance and obesity via Tlr4/Ikbke in mice

**DOI:** 10.1038/s41598-020-67040-3

**Published:** 2020-06-23

**Authors:** Clara Machado Campolim, Lais Weissmann, Clílton Kraüss de Oliveira Ferreira, Olivia Pizetta Zordão, Ana Paula Segantine Dornellas, Gisele de Castro, Tamires Marques Zanotto, Vitor Ferreira Boico, Paula Gabriele Fernandes Quaresma, Raquel Patrícia Ataíde Lima, Jose Donato, Mariana Matera Veras, Paulo Hilário Nascimento Saldiva, Young-Bum Kim, Patricia Oliveira Prada

**Affiliations:** 1 School of Applied Sciences, State University of Campinas (UNICAMP), Limeira, SP Brazil; 2 Department of Internal Medicine, School of Medical Science, State University of Campinas (UNICAMP), Campinas, SP Brazil; 3 Department of Physiology and Biophysics, Institute of Biomedical Sciences, University of São Paulo (USP), São Paulo, SP Brazil; 4 Laboratório de Poluição Atmosférica Experimental LIM05, Hospital das Clínicas HCFMUSP, Faculdade de Medicina, Universidade de São Paulo (USP), São Paulo, SP Brazil; 5 Division of Endocrinology, Diabetes and Metabolism, Department of Medicine, Beth Israel Deaconess Medical Center, Harvard Medical School, Boston, MA USA

**Keywords:** Atmospheric science, Risk factors, Chronic inflammation, Hypothalamus, Obesity

## Abstract

A previous study demonstrated that a high-fat diet (HFD), administered for one-three-days, induces hypothalamic inflammation before obesity’s established, and the long term affects leptin signaling/action due to inflammation. We investigate whether exposure to particulate matter of a diameter of ≤2.5 μm (PM_2.5_) in mice fed with a chow diet leads to similar metabolic effects caused by high-fat feeding. Compared to the filtered air group (FA), one-day-exposure-PM_2.5_ did not affect adiposity. However, five-days-exposure-PM_2.5_ increased hypothalamic microglia density, toll-like-receptor-4 (Tlr4), and the inhibitor-NF-kappa-B-kinase-epsilon (Ikbke) expression. Concurrently, fat mass, food intake (FI), and ucp1 expression in brown adipose tissue were also increased. Besides, decreased hypothalamic STAT3-phosphorylation and Pomc expression were found after twelve-weeks-exposure-PM_2.5_. These were accompanied by increased FI and lower energy expenditure (EE), leading to obesity, along with increased leptin and insulin levels and HOMA. Mechanistically, the deletion of Tlr4 or knockdown of the Ikbke gene in the hypothalamus was sufficient to reverse the metabolic outcomes of twelve-weeks-exposure-PM_2.5_. These data demonstrated that short-term exposure-PM_2.5_ increases hypothalamic inflammation, similar to a HFD. Long-term exposure-PM_2.5_ is even worse, leading to leptin resistance, hyperphagia, and decreased EE. These effects are most likely due to chronic hypothalamic inflammation, which is regulated by Tlr4 and Ikbke signaling.

## Introduction

The most harmful fraction of air pollution is the airborne particulate matter with a diameter of 2.5 μm or less (PM_2.5_), which is small enough to penetrate the tissues through the lungs and olfactory epithelium^1^, leading to several diseases and even death^2–4^. There is causal evidence linking chronic exposure to PM_2.5_ and the development of metabolic diseases, including obesity and type 2 diabetes mellitus^4–7^. The inflammatory response has been the mechanism proposed as the link between chronic exposure to PM_2.5_ and metabolic diseases^1,8–11^.

The high palatable diet is associated with inflammation in peripheral tissues and hypothalamic nuclei, the latter being involved in the regulation of energy homeostasis^12–15^. A previous study showed that one day on a high-fat diet (HFD) was sufficient to trigger an inflammatory response in the hypothalamus. It occurred before changes in body adiposity^15^, suggesting that inflammatory markers are probably implicated in the development of obesity. Since exposure to PM_2.5_ also triggers inflammation in the peripheral tissues and hypothalamus^10,11,16,17^, by analogy, we asked if mice exposed to PM_2.5_ for a short-term while receiving chow diet could also induce this effect before obesity was developed.

The hypothalamus is responsible for maintaining energy homeostasis by integrating neural, nutritional, and hormonal signals^12,14^. In particular, leptin receptor-expressing neurons (LEPR) in the hypothalamic nuclei are critical for regulating food intake (FI) and energy expenditure (EE)^12,14^. Leptin begins its intracellular signal by engaging LEPR and activating the JAK2/STAT3 (Janus kinase 2)/(signal transducer and activator of transcription 3) pathway, which is crucial for the control of food ingestion^12,14^. Leptin signaling through pro-opiomelanocortin (Pomc) neurons promotes a striking anorexigenic effect while increasing EE. In contrast, leptin inhibits neurons co-expressing the LEPR and potent inducers of food intake such as agouti-related protein (Agrp), neuropeptide Y (Npy) and the neurotransmitter gamma-aminobutyric acid (GABA)^12,14^.

Chronic high fat feeding induces enlargement of white adipose tissue deposits and a state of low-grade inflammation^12,14^. Toll-like receptor 4 (TLR4) is known to contribute to insulin resistance and obesity^18–20^. The canonical pathway used by TLR4 signaling depends on the myeloid differentiation factor of adapter protein 88 (MyD88). In its turn, MyD88 activates the signaling pathway of the inhibitor of nuclear factor kappa-B kinase subunit beta (IKKβ) and the nuclear-kappa B transcription factor (NFκB) increasing pro-inflammatory gene expression^20–23^. TLR4 can also signal via a non-canonical (or MyD88-independent) pathway responsible for the recruitment of Tank 1 binding kinase and IKK epsilon subunit (IKKε), which increases interferon type I levels and triggers innate and adaptive immune responses^24,25^. Deletion or inhibition of TLR4 or MyD88 from the hypothalamus restores energy homeostasis in rodents receiving HFD^20,21,26^. However, both IKKβ and IKKε are upregulated in the hypothalamus of HFD-fed mice^27,28^. Excluding or eliminating only one of them is sufficient to restore leptin signaling, allowing energetic homeostasis^27,28^.

Previously studies have shown that the inhibition of tumor necrosis factor-alpha (TNF-α) or the use of the IKKβ inhibitor in the hypothalamus of mice chronically exposed to PM_2.5_ reduce inflammation in peripheral tissues^16,17,29^. The above studies suggest a link between hypothalamic inflammation induced by PM_2.5_ exposure and metabolic dysfunction. However, it remains unknown whether prolonged exposure to PM_2.5_ could attenuate leptin signaling/action as this hormone is well characterized as the principal regulator of the energy balance involved in the control of food intake and energy expenditure.

Based on the evidence discussed above, our aims were to investigate in mice under a normal chow diet: (1) whether short-term (one to five days) exposure to PM_2.5_ induces hypothalamic inflammation before the development of obesity as occurs in mice fed a HFD; (2) whether long-term (12 weeks) exposure to PM_2.5_ impairs leptin signaling/action in the hypothalamus, contributing to an increase of food intake and decrease of energy expenditure, and (3) whether inflammatory mediators such as TLR4 and IKKε play a role in impairing leptin signaling and action.

## Results

### Short-term exposure to PM_2.5_ triggers hypothalamic inflammation and increases fat mass and food intake

**Figure 1:**
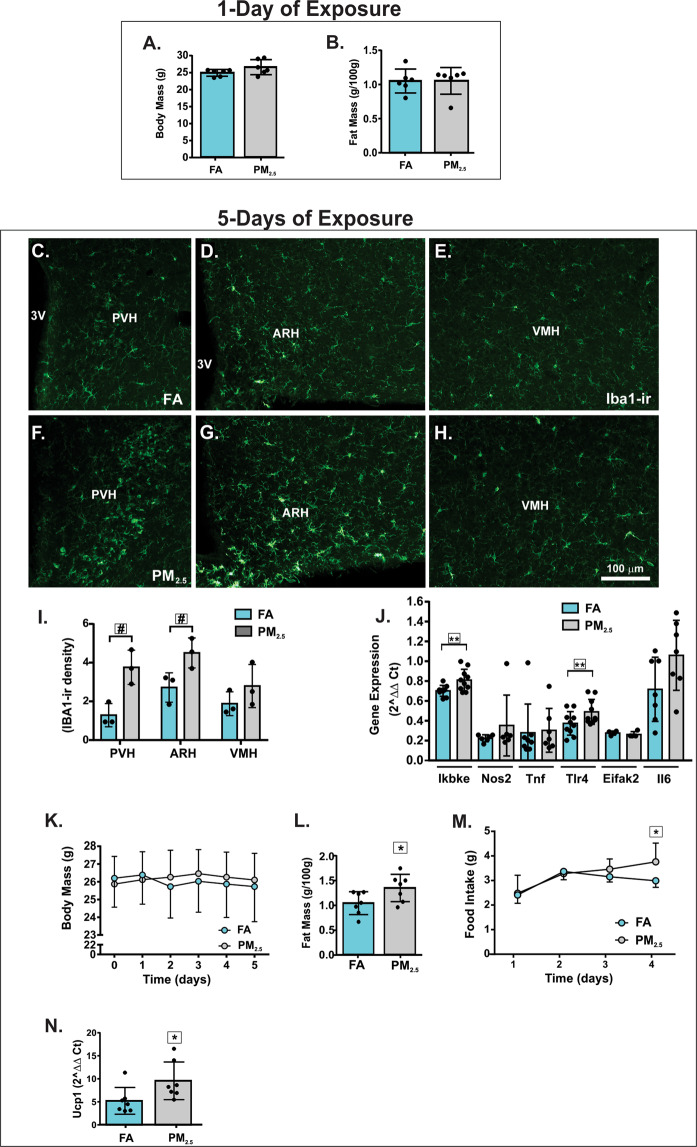
Progressive exposure to PM_2.5_ increases fat mass and food intake and triggers hypothalamic inflammation. Mice were exposed to particulate matter less than 2.5 micrometers in diameter (PM_2.5_) or filtered air (FA) for 1 day or 5 days, as indicated in the Fig. (**A**) body mass (g) n = 6 for each group. (**B**) Fat mass (g/100 g of body mass) n = 6 for each group. Epifluorescence photomicrographs showing Iba1-immunoreactivity (Iba1-ir) in the hypothalamamic nuclei of mice exposed to FA (**C to E**) or PM_2.5_ (**F to H**) for 5 days n = 3 for each group. (**I**) Iba-1-ir density expression in hypothalamic nuclei of mice exposed to FA or PM_2.5_ for 5 days. (**J**) Hypothalamic gene expression of inflammatory markers from mice exposed to PM_2.5_ for 5 days n = 4–10. (**K)** body mass (g) n = 7 for each group. (**L)** Fat mass (g/100 g of body mass) n = 7 for each group. (**M**) Food intake (g) n = 7 for each group. (**N**) *Ucp1* gene expression in the brown adipose tissue (BAT) (2^ΔΔ Ct) n = 7 for each group. Abbreviations: 3 V, third ventricle; ARH, arcuate nucleus; PVH, paraventricular nucleus; VMH, ventromedial nucleus of the hypothalamus; Ikbke - inhibitor of nuclear factor kappa-B kinase subunit epsilon; Nos2 - inducible nitric oxide synthase 2; Tnf - Tumor necrosis factor alpha; Tlr4 - Toll-like receptor 4; Eifak2 - Eukaryotic translation initiation factor-α kinase 2; Il6 - Interleukin 6. All of the mice studied were 6–8 weeks of age. Data were presented as the mean ± SD. Unpaired t test (two-tailed) was used for the statistical analysis of Panels *A, B, D* and *F* and two-way ANOVA followed by the Tukey post hoc test was used for statistical analysis of Panels *C* and *E*. *P < 0.05 vs FA, ^#^P < 0.05 vs FA (same hypothalamic nuclei), **P < 0.05 vs FA (same gene).

After one day of exposure to PM_2.5_ or FA, body mass and epididymal fat mass was not significantly different between the groups (Fig. 1A,B). The levels of pro-inflammatory cytokines were not different between the groups (data not shown). However, mice exposed to PM_2.5_ for five days exhibited a significant increase of Iba1 staining in the paraventricular nucleus (PVH) and in the arcuate nucleus of the hypothalamus (ARH), but not in the ventromedial nucleus of the hypothalamus (Fig. 1C–I). The levels of Ikbke and Tlr4 gene expression were increased in the hypothalamus after five days of PM_2.5_ exposure whereas no statistically significant difference was observed in Nos2, Eifak2, and Il6 gene expression (Fig. 1J). After five days, PM_2.5_-exposed mice had an increase in epididymal fat mass without changing the body mass significantly compared to the FA-exposed mice (Fig. 1K,L). Food intake slightly increased on the fourth day of exposure to PM_2.5_ (Fig. 1M). Surprisingly, the expression of the uncoupling protein 1 (Ucp1) gene in brown adipose tissue (BAT) was elevated in mice exposed to PM_2.5_ compared to mice exposed to FA (Fig. 1N). The levels of corticosterone (ηg/mL) were not significantly different between the groups (FA: 7.91 ± 3.08, n = 5; PM_2.5_: 7.63 ± 2.58, n = 5; p = 0.8808).

### Long-term exposure to PM2.5 leads to hyperphagia, reduced EE and obesity

**Figure 2:**
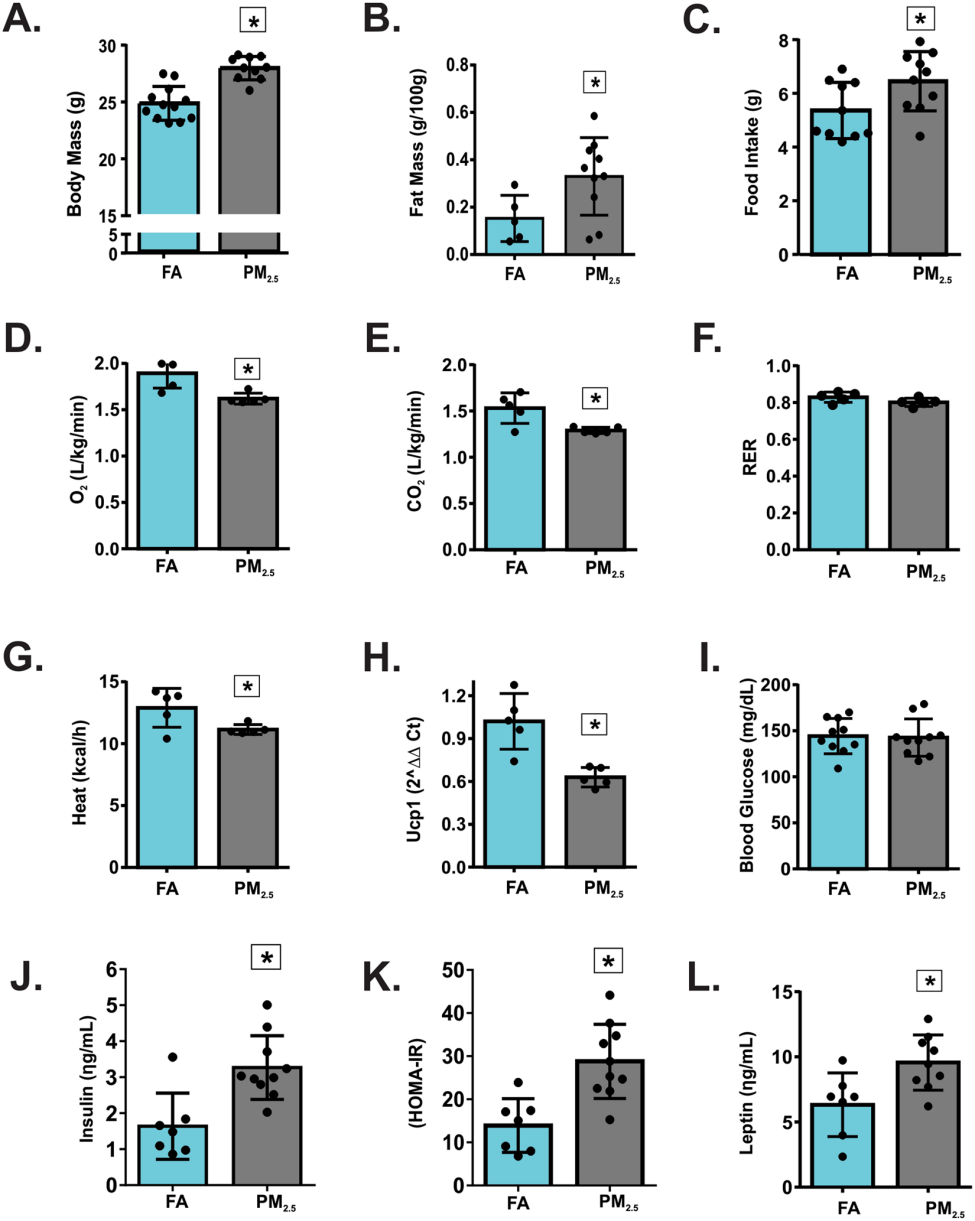
Long-term exposure to PM_2.5_ leads to hyperphagia, reduced EE and obesity. Mice were exposed to particulate matter less than 2.5 micrometers in diameter (PM_2.5_) or filtered air (FA) for 12 weeks. (**A)** Body mass (g) n = 12 for FA group and n = 10 for PM_2.5_ group. (**B)** Fat mass (g/100 g of body mass) n = 5 for FA group and n = 10 for PM_2.5_ group. (**C)** Food intake (g) n = 10 for each group. (**D)** O_2_ consumption (L/kg/min) n = 5 for each group. (**E)** CO_2_ production (L/kg/min) n = 5 for each group. (**F)** Respiratory exchange ratio (RER) n = 5 for each group (**G)** Heat production (kcal/h) n = 5 for each group. (**H)** *Ucp1* gene expression in the brown adipose tissue (BAT) (2^ΔΔ Ct) n = 5 for each group. (**I)** fasting blood glucose (mg/dL) n = 10 for each group. (**J)** Fasting serum insulin (ηg/mL) n = 7 for FA group and n = 10 for PM_2.5_ group. (**K)** Homeostasis model assessment **(**HOMA-IR) index n = 7 for FA group and n = 10 for PM_2.5_ group. (**L)** Fasting serum leptin levels (ηg/mL) n = 7 for FA group and n = 9 for PM_2.5_. All of the mice studied were 6–8 weeks of age. Data were presented as the mean ± SD. Unpaired t test (two-tailed) was used for the statistical analysis *P < 0.05 vs FA.

A marked increase in body mass and epididymal fat mass was observed after 12 weeks of exposure to PM_2.5_ (Fig. 2A,B). Food intake increased, and O_2_ consumption and CO_2_ production decreased in PM_2.5_-exposed mice compared to the FA-exposed mice (Fig. 2C–E). Respiratory Exchange Ratio (RER) was not significantly different between the groups (Fig. 2F). PM_2.5_-exposed mice had significantly decreased heat production and Ucp1 gene expression in BAT compared to the FA-exposed group (Fig. 2G,H). The levels of corticosterone (ηg/mL) were not significantly different between the groups (FA: 8.92 ± 3.50, n = 5; PM_2.5_: 8.37 ± 2.30, n = 5; p = 0.7747). Fasting blood glucose levels were not significantly different between the groups; however, PM_2.5_-exposed mice had a significant increase in insulin levels compared to the FA-exposed mice (Fig. 2I,J). Accordingly, the Homeostasis Model Assessment Index (HOMA-IR) was increased in the PM_2.5_-exposed mice (Fig. 2K). The exposure to PM_2.5_ significantly increased the levels of leptin compared to the FA-exposed mice (Fig. 2L).

### Long-term exposure to PM2.5 impairs leptin sensitivity, alters neuropeptides expression and increases inflammatory mediators

**Figure 3:**
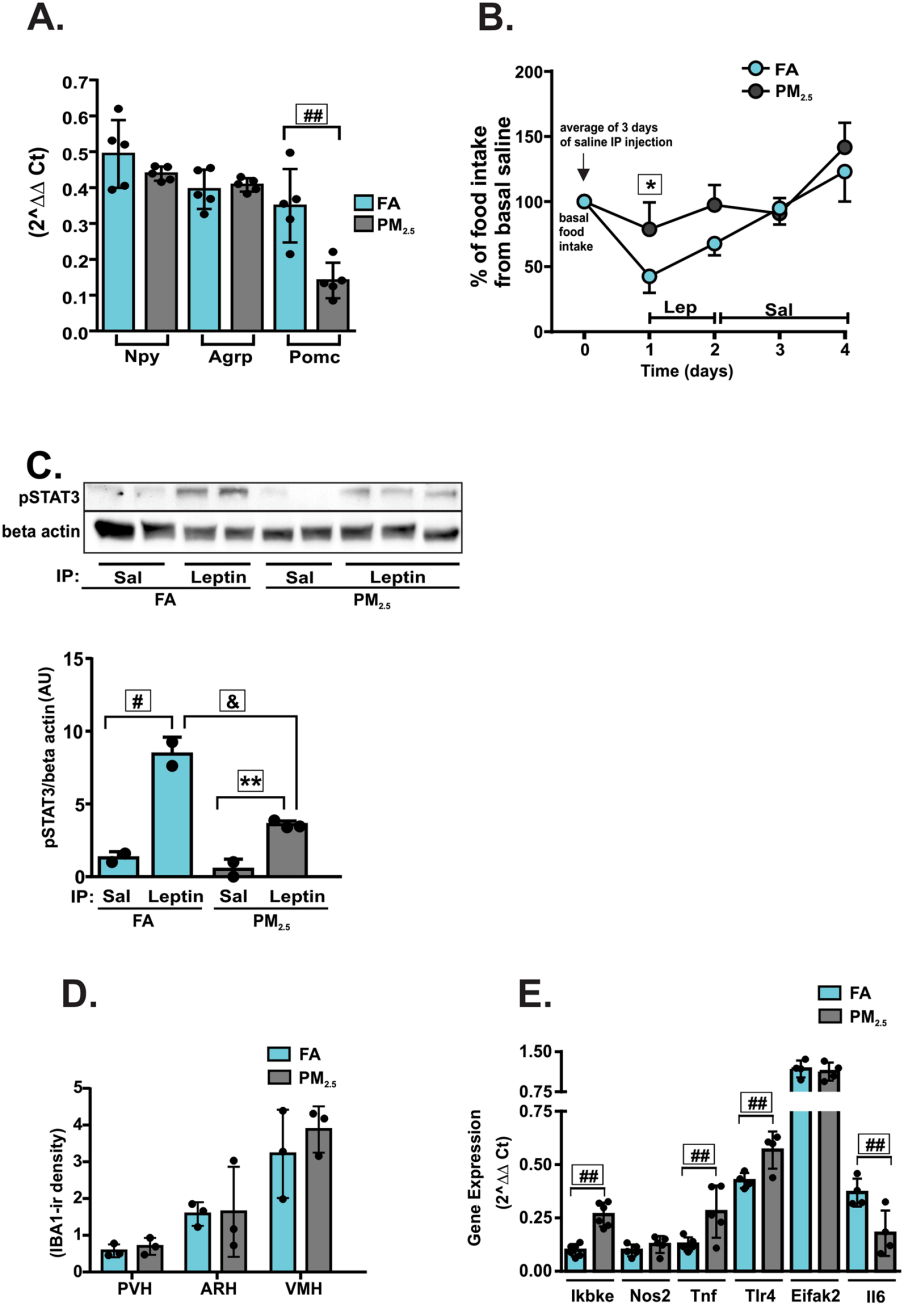
Long-term exposure to PM_2.5_ impairs leptin sensitivity, alters the expression of hypothalamic neuropeptides and increases inflammatory mediators. Mice were exposed to particulate matter less than 2.5 micrometers in diameter (PM_2.5_) or filtered air (FA) for 12 weeks. (**A)** Npy (neuropeptide Y), Agrp (Agouti-related protein) and Pomc (Pro-opiomelanocortin) gene expression in the hypothalamus after 24 h of fasting n = 5 for each group. (**B)** Intraperitoneal (IP) leptin sensitivity test, the results of food intake during the 3 days of leptin injection was compared to the basal FI for each mouse and were expressed as a percent of the basal (saline) food intake for each mouse n = 4 for each group. (**C)** STAT3 phosphorylation (Arbitrary Units) in response to IP leptin or saline injection was measured in the hypothalamus of overnight fasted mice; n = 2 for FA (saline, leptin) and PM_2.5_ (saline) groups and n = 3 for PM_2.5_ (leptin) group (full-length gels are presented in Supplementary Fig. 1A). (**D)** Iba-1-ir density expression in hypothalamic nuclei and (**E)** hypothalamic gene expression of mice exposed to FA or PM_2.5_ for 12 weeks n = 4–6. Abbreviations: Ikbke - inhibitor of nuclear factor kappa-B kinase subunit epsilon; Nos2 - inducible nitric oxide synthase 2; Tnf - Tumor necrosis factor alpha; Tlr4 - Toll-like receptor 4; Eifak2 - Eukaryotic translation initiation factor-α kinase 2; Il6 - Interleukin 6. All of the mice studied were 6–8 weeks of age. Data were presented as the mean ± SD. One-way ANOVA (Panels *A* and *C*) and two-way ANOVA (Panel *B*) followed by a Tukey post hoc test were used for the statistical analysis *P < 0.05 vs FA; #*P* < 0.05 vs. FA leptin; ^&^*P* < 0.05 vs. PM_2.5_ leptin; **P < 0.05 vs PM_2.5_ leptin; ^##^P < 0.05 vs FA (same gene).

The gene expression levels of Npy or Agrp in the hypothalamus were not significantly different between the groups. However, PM_2.5_-exposed mice had a significantly decreased Pomc expression compared to the group of mice exposed to FA (Fig. 3A). PM_2.5_-exposed mice had a significantly decreased anorexigenic response to leptin when compared to the FA-exposed group (Fig. 3B), suggesting leptin resistance. To further characterize the resistance to leptin, STAT3 phosphorylation in the hypothalamus was analyzed in both groups. Leptin injection increased STAT3 phosphorylation in the hypothalamus of the FA group compared to saline-injected mice of the FA group. Mice exposed to PM_2.5_ also showed increased STAT3 phosphorylation in the hypothalamus in response to leptin compared to saline-injected mice of the PM_2.5_-exposed group. However, the magnitude of this response was attenuated when compared to the phosphorylation levels detected in the group of mice exposed to FA (Fig. 3C; Supplementary Fig. 1A). After 12 weeks of PM_2.5_ exposure, Iba1 staining in the PVH, ARH, and VMH was not different from mice exposed to FA (Fig. 3D). Nevertheless, after 12 weeks of exposure to PM_2.5_, Ikbke and Tlr4 gene expression remained increased, while Tnf gene expression increased and IL-6 levels decreased. No statistically significant differences were observed in Nos2, and Eifak2 gene expressions (Fig. 3E). Considering the above results, we hypothesized that a non-canonical pathway involving Tlr4 and Ikbke could be responsible for the development of inflammation and leptin resistance. To investigate this hypothesis, we repeated the experiments in TLR4^−/−^ mice exposed to FA or PM_2.5_.

### Deletion of TLR4 protected mice from leptin resistance and obesity induced by chronic exposure to PM2.5

**Figure 4:**
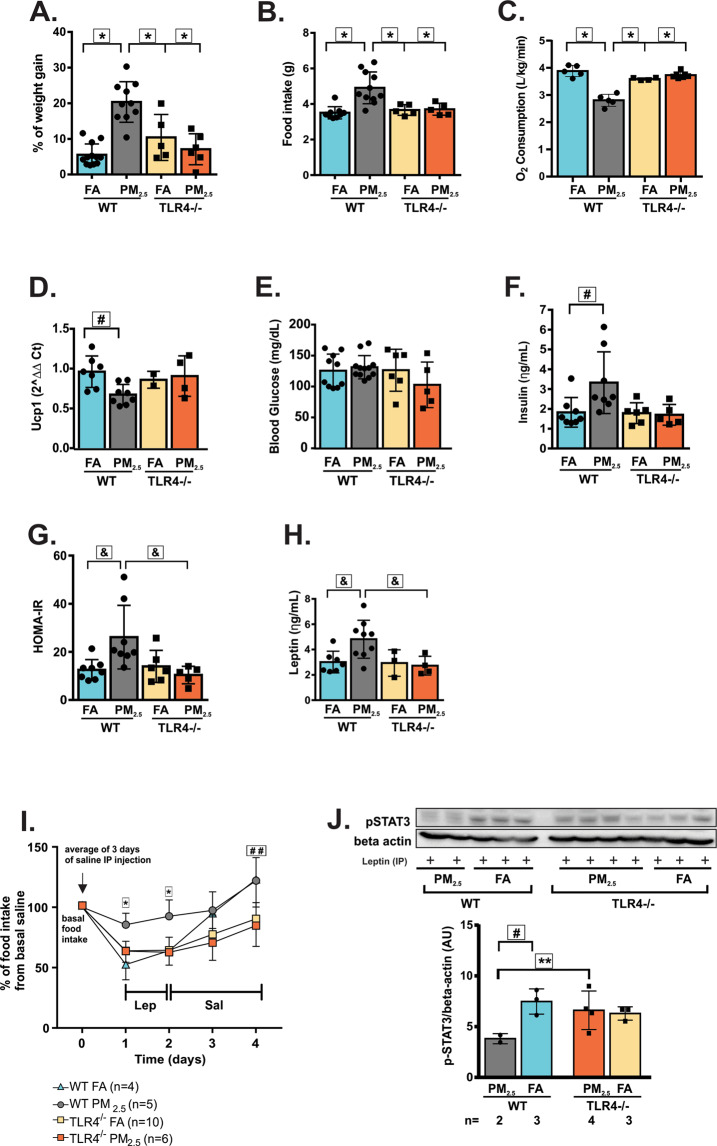
Deletion of TLR4 protected mice from leptin resistance and obesity induced by chronic exposure to PM_2.5_. TLR4^−/−^ and WT mice were exposed to particulate matter less than 2.5 micrometers in diameter (PM_2.5_) or filtered air (FA) for 12 weeks. (**A)** Percent of weight gain from the beginning of exposures. (**B)** Food intake (g). (**C**) O_2_ consumption (L/kg/min). (**D)** Ucp1 gene expression in the brown adipose tissue (**E)** Fasting blood glucose (mg/dL). (**F)** Fasting serum insulin (ηg/mL). (**G)** Homeostasis model assessment **(**HOMA-IR) index. (**H)** Fasting serum leptin levels (ηg/mL). (**I)** Intraperitoneal (IP) leptin sensitivity test. (**J)** STAT3 phosphorylation (Arbitrary Units) in response to IP leptin injection was measured in the hypothalamus of overnight fasted mice (full-length gels are presented in Supplementary Fig. 1B). All of the mice studied were 6–8 weeks of age. Data were presented as the mean ± SD. Two-way ANOVA (considering the genotypes and exposures as 2 different factors) followed by a post hoc test were used for the statistical analysis. *P < 0.05 vs WT FA, TLR4^−/−^ FA and PM_2.5_; ^#^*P* < 0.05 vs WT FA; &p < 0.05 vs WT FA and TLR4^−/−^ PM_2.5_; ^##^*P* < 0.05 vs TLR4^−/−^ FA and PM_2.5_; **P < 0.05 vs TLR4^−/−^ PM_2.5_. **Letters A–H**: WT FA n = 5–11; WT PM_2.5_ n = 5–13; TLR4^−/−^ FA n = 2–6; TLR4^−/−^ PM_2.5_ n = 4–7.

To investigate the contribution of TLR4 in the development of obesity, TLR4^−/−^ mice were exposed to PM_2.5_ or FA for 12 weeks. The experiments were also conducted in WT mice. WT exposed to PM_2.5_ developed increased weight gain after chronic exposure to PM_2.5_ compared to WT exposed to FA. The deletion of TLR4 protected mice from weight gain since WT mice exposed to PM_2.5_ developed enhanced weight gain compared to TLR4^−/−^ mice exposed to FA or PM_2.5_ (Fig. 4A). The deletion of TLR4 also protected mice from hyperphagia since WT mice exposed to PM_2.5_ ate more compared to TLR4^−/−^ mice exposed to FA or PM_2.5_ (Fig. 4B). WT exposed to PM_2.5_ had reduced O_2_ consumption compared to WT FA-exposed mice and TLR4^−/−^ exposed to FA or PM_2.5_ (Fig. 4C). Ucp1 gene expression in BAT was decreased in the WT exposed to PM_2.5_ compared to WT FA-exposed mice (Fig. 4D). There was no significant difference among the four groups in fasting blood glucose levels (Fig. 4E). However, compared to WT mice that were exposed to FA, WT PM_2.5_-exposed mice showed higher fasting insulin levels (Fig. 4F). Probably due to higher insulin levels, WT exposed to PM_2.5_ exhibited higher HOMA-IR compared to WT FA-exposed mice. Besides, HOMA-IR was elevated in WT mice exposed to PM_2.5_ compared to TLR4^−/−^ mice exposed to PM_2.5_ (Fig. 4G). Fasting leptin levels were significantly increased in the WT PM_2.5_-exposed group compared to both WT FA-exposed mice and TLR4^−/−^ PM_2.5_-exposed mice (Fig. 4H). Leptin sensitivity test demonstrated that at the first and second days of leptin injection WT PM_2.5_-exposed mice exhibited a significantly decreased anorexigenic response to leptin compared to other groups. This response was abolished after the beginning of saline injection. At the end of the experiment TLR4^−/−^ either exposed to FA and PM_2.5_ were eating less than WT exposed to FA and PM_2.5_ (Fig. 4I). To further characterize the resistance to leptin, STAT3 phosphorylation in the hypothalamus was determined in all groups. Compared to WT mice that were exposed to PM_2.5_, WT FA-exposed mice and TLR4^−/−^ PM_2.5_-exposed mice exhibited increased STAT3 phosphorylation in response to leptin (Fig. 4J; Supplementary Fig. 1B). There was no significant difference among the four groups in corticosterone levels (ηg/mL) (WT FA: 9.19 ± 2.38, n = 5 vs. WT PM_2.5_: 9.11 ± 2.40, n = 5; TLR4^−/−^ FA: 8.85 ± 2.79, n = 5 vs. TLR4^−/−^ PM_2.5_: 8.41 ± 1.23, n = 5, Two-Way ANOVA: p = 0.8646).

### Specific knocking down of hypothalamic Ikbke protected mice from obesity, and hypothalamic leptin resistance induced by chronic exposure to PM2.5

**Figure 5:**
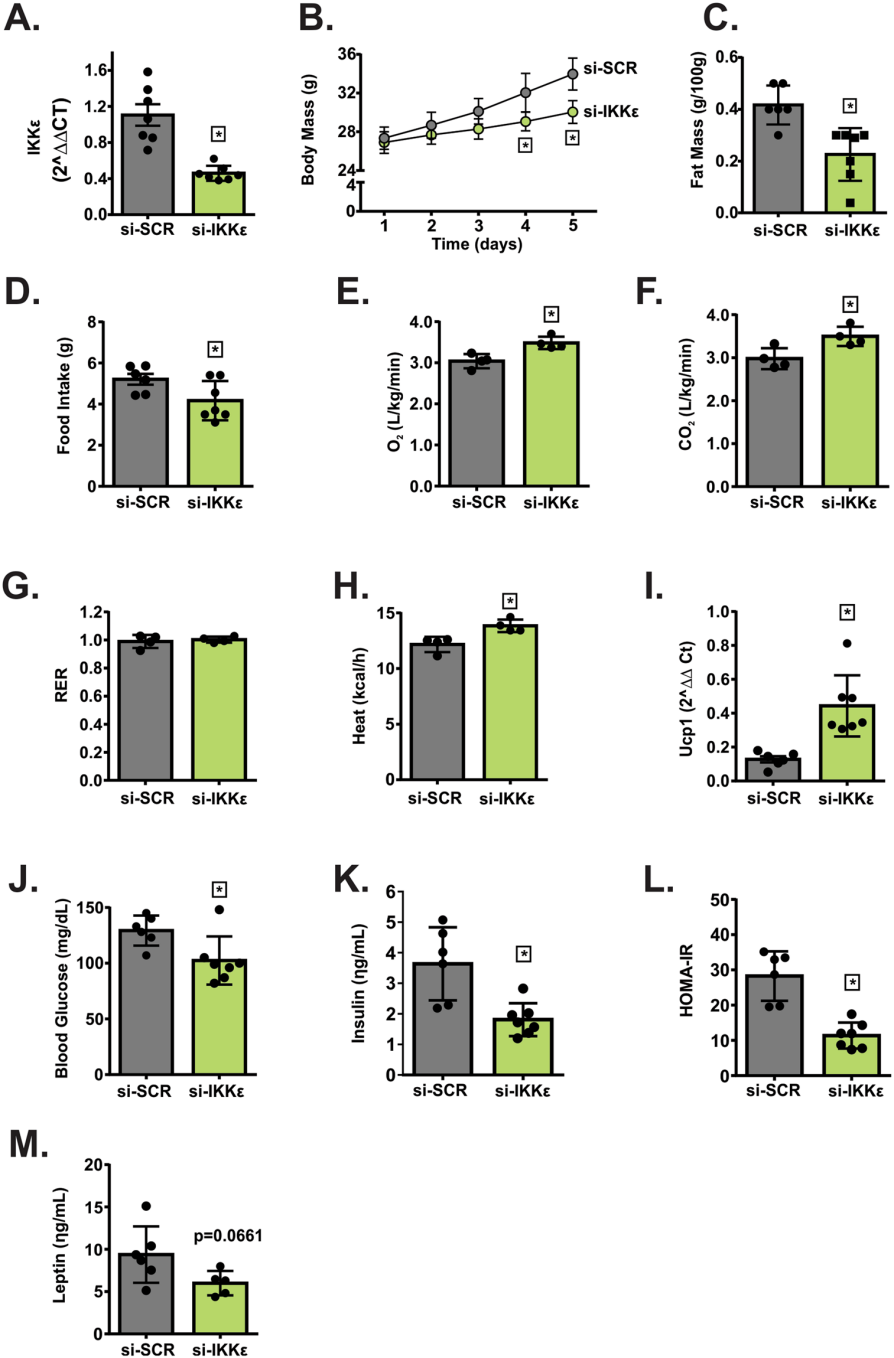
Specific knocking down of hypothalamic Ikbke protected mice from obesity and insulin resistance induced by chronic exposure to PM_2.5._ To deeper assess the role of non-canonical TLR4 pathway in the development of obesity and insulin resistance, in WT-exposed mice Ikbke was knocked down in the hypothalamus before the final week of exposure. WT exposed to PM_2.5_ were treated with small interfering RNA against IKKε (si-IKKε)^28^ or si-SCR (control). (**A)** Ikbke gene expression in the hypothalamus of mice n = 7 each group. (**B)** Body mass (g), (**C**) Fat mass (g/100 g of body mass). (**D)** Food intake (g). (**E)** O_2_ consumption (L/kg/min), (**F)** CO_2_ production (L/kg/min), (**G)** Respiratory exchange ratio (RER) and (**H)** Heat (kcal/h) n = 4 for each group. (**I)** *Ucp1* gene expression in the brown adipose tissue (**J)** fasting blood glucose (mg/dL). (**K)** fasting serum insulin (ηg/mL). (**L)** Homeostatic model assessment (HOMA-IR) index and (**M)** fasting serum leptin levels (ηg/mL). Panels *B, C*, and *J-M* had n = 6–7. Data were presented as mean ± SD from random mice at 8–10 weeks of age. Unpaired t test (two-tailed) was used for the statistical analysis of Panels *A, C-M*. Two-way ANOVA followed by a Tukey post hoc test was used for the statistical analysis of Panel *B*. *P < 0.05 vs si-SCR group.

**Figure 6:**
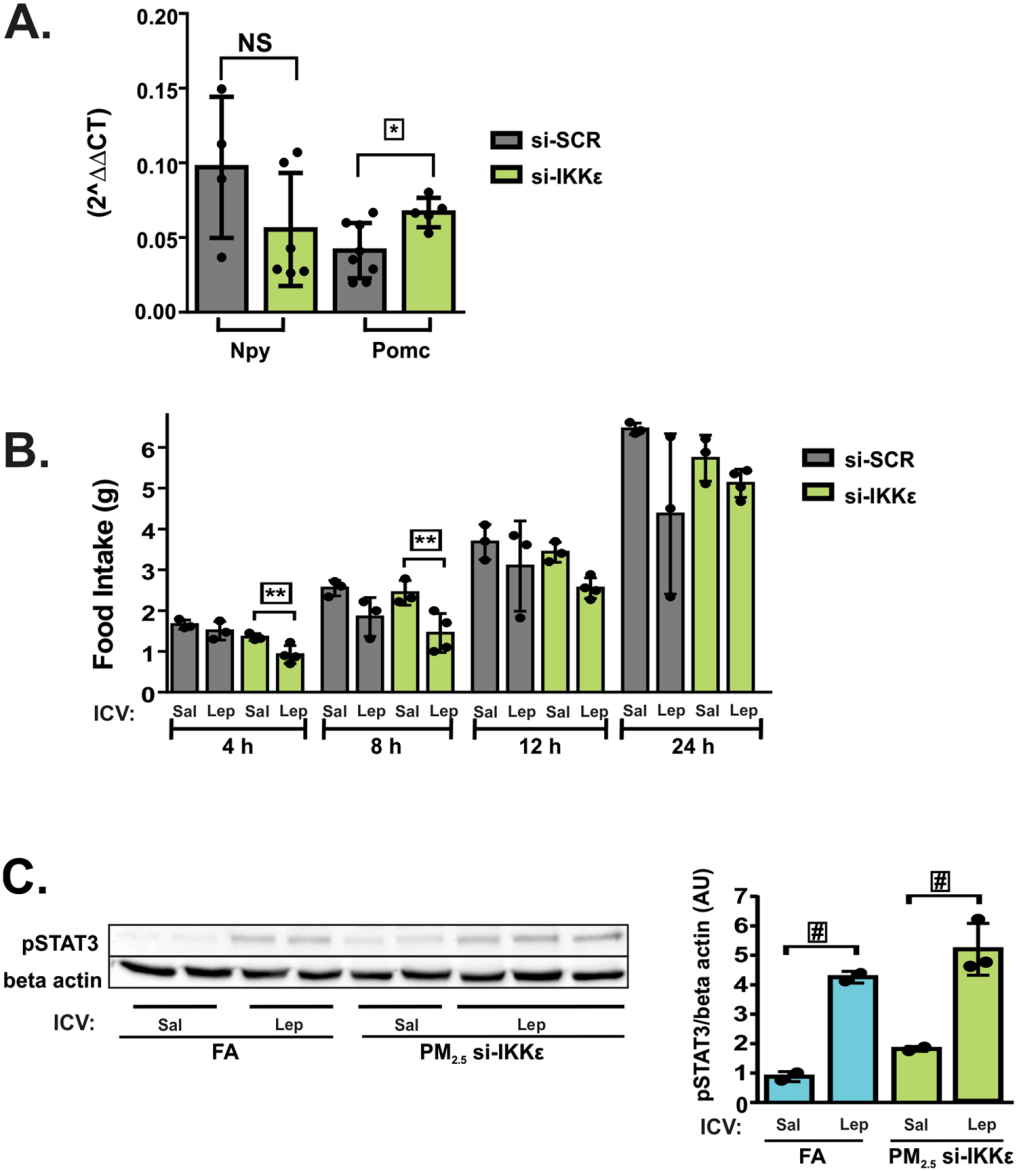
Specific knocking down of hypothalamic Ikbke reversed leptin resistance induced by chronic exposure to PM_2.5._ WT exposed to PM_2.5_ were treated with small interfering RNA against IKKε (si-IKKε) or si-SCR (control). (**A)** Npy (neuropeptide Y), and Pomc (Pro-opiomelanocortin) gene expression in the hypothalamus after 24 h of fasting n = 4–8. (**B)** For ICV leptin sensitivity test, mice already cannulated underwent to overnight fasting prior to leptin or saline ICV injections. The concentration of leptin was 10 ηg/µL as previously validated^28^. The volume infused for both leptin and saline was 1 µL. Then, cumulative food intake was measured for 4, 8, 12 and 24 h n = 3–4. (**C)** STAT3 phosphorylation (Arbitrary Units) in response to ICV leptin or saline injection was measured in the hypothalamus of overnight fasted mice; FA saline and leptin: n = 2; PM_2.5_ si-SCR: n = 2 and PM_2.5_ si-IKKε: n = 3 (full-length gels are presented in Supplementary Fig. 2C). All of the mice studied were 6–8 weeks of age. Data were presented as the mean ± SD. One-way ANOVA followed by a Tukey post hoc test was used for the statistical analysis *P < 0.05 vs si-SCR; **P < 0.05 si-IKKε saline vs. si-IKKε leptin; ^#^*P* < 0.05 vs. same group with saline.

To deeper assess the role of non-canonical TLR4 pathway in the development of obesity and leptin resistance, in WT-exposed mice Ikbke was knocked down in the hypothalamus before the final week of exposure. The si-IKKε marked reduced (50%) the expression of Ikbke in the medial basal hypothalamus (MBH) of mice exposed to PM_2.5_ and treated with si-SCR (Fig. 5A). No difference was observed in the Ikbke expression among WT FA treated with either si-IKKε or si-SCR and WT PM_2.5_-treated with si-SCR (data not shown). This result probably occurred due to the increased Ikbke expression only in the MBH of animals exposed to PM_2.5_. Therefore, we continue the other experiments using the WT mice exposed to PM_2.5_ and treated with si-SCR or si-RNA-IKKε. Body mass decreased from the fourth day of si-RNA-IKKε infusion and persisted up to the fifth day, accompanied by a reduction of epididymal fat mass (Fig. 5B,C). The infusion of si-RNA-IKKε significantly decreased the food intake while increased the O_2_ consumption, CO_2_ production and Heat compared to si-RNA-SCR-infused mice. There was no significant difference between the groups in RER (Fig. 5D,H). Similarly to the result of O_2_ consumption, the infusion of si-RNA-IKKε significantly increased the expression of Ucp1 in BAT (Fig. 5I). Blood glucose and insulin levels as well as HOMA-IR were reduced in animals infused with si-RNA-IKKε (Fig. 5J–L). Fasting leptin levels tended to be decreased (p = 0.0661) in mice infused with si-RNA-IKKε (Fig. 5M). No difference was observed in the Npy gene expression between groups. However, animals infused with si-RNA-IKKε had a significantly increased Pomc expression compared to the group of mice infused with si-SCR (Fig. 6A). The infusion of si-RNA-IKKε significantly decreased the food intake in response to leptin after 4 to 8 h from injection, suggesting an improvement of leptin action after IKKε inhibition (Fig. 6B). Accordingly, leptin injection increased STAT3 phosphorylation in the hypothalamus of the FA group compared to saline-injected mice of the FA group. Mice exposed to PM_2.5_ and infused with si-RNA-IKKε also showed increased STAT3 phosphorylation in response to leptin compared to saline-injected mice of the si-RNA-IKKε group (Fig. 6C; Supplementary Fig. 2A), suggesting that the inhibition of IKKε reversed leptin resistance induced by the exposure to PM_2.5_.

## Discussion

The findings presented here demonstrated that five days of exposure to PM_2.5_ was sufficient to induce hypothalamic inflammation before the development of obesity, similar to what occurs in mice fed with a HFD^13,15^. These effects are required for hypothalamic TLR4/IKK signaling. Likewise, rodents fed with a western diet for ten days displayed increased inflammatory activity induced by c-jun N-terminal kinase (JNK) in the hypothalamus without changes in body mass^13^.

In this study, we found higher food intake in animals exposed to PM_2.5_ for five days, in the same way that was observed in rodents on a high-fat diet for one to seven days and in rodents on a western diet for ten days^15^. The inflammation that occurs in the hypothalamus caused by pollution or altered food composition is the primary cause of hyperphagia observed in the present study and in other studies^13,15^.

It is evident that ucp1 is a key determinant of BAT thermogenic activity^30^. Our data that mice exposed to PM_2.5_ for five days presented increased ucp1 expression in BAT suggest a possible increase in the thermogenesis. Given the fact that interscapular BAT is tightly innervated by the sympathetic nerve system that stimulates thermogenesis via the β3AR^31,32^, it is conceivable that hypothalamic inflammation caused by pollution may affect the activity of the sympathetic activity via an unidentified mechanism, which could regulate UCP1 expression in BAT. Future investigation will be warranted for this issue.

Ikbke and Tlr4 gene expression remained elevated in the hypothalamus of mice exposed to PM_2.5_ for 12 weeks. Tnf gene expression was also increased concurrently, suggesting a progression of the inflammatory process, because Tnf is a cytokine responsible for stimulating the production of other pro-inflammatory cytokines favoring the development of chronic inflammation^33,34^. An impairment of leptin action and signaling, as well as lower Pomc gene expression, was detected in mice exposed to PM_2.5_ for 12 weeks, leading to energy imbalance. Considering that the deletion of Tlr4 or a specific knocking-down of Ikbke in the hypothalamus was sufficient to reverse the obese phenotype induced by chronic exposure to PM_2.5_, it is likely that the inflammatory milieu is involved in the disruption of leptin signals. Taking together, these data suggest that chronic air pollution exposure triggers hypothalamic inflammation, which disrupts satiety signals being a risk factor for the development of obesity and its associated comorbidities.

Particulate matter is a key contributor to inducing inflammatory diseases^1,35,36^. Although it can be ingested, the passage of PM_2.5_ through the nasal route is the recognized entrance portal^37,38^. Through this route, the development of local and systemic inflammation occurs involving the release of pro-inflammatory mediators such as IL-1β, among others contributing to neurodegenerative diseases such as Alzheimer’s and Parkinson’s diseases^39–41^. Another way to incorporate the particulate matter, especially the ultrafine fractions, is through neurons in the olfactory epithelium, increasing the inflammatory response and potentially disrupting the blood-brain barrier^37–39,41^. In this study, we think that the main entry of PM_2.5_ should be via neurons in the olfactory epithelium, directly accessing the central nervous system. This is supported by the fact that inflammatory markers in the hypothalamus are increased a few days after exposure to PM_2.5_.

In the present study, hypothalamic inflammation was observed shortly after five days of exposure to PM_2.5_ and before major changes in the energy metabolism, suggesting that hypothalamic inflammation may be occur first. Similar results were observed in mice fed with a high-fat diet for a short period, in which 1 to 3 days in this diet were sufficient to trigger hypothalamic inflammation and neuronal injury before the rodents gained more adiposity^15^. There are more similarities between hypothalamic inflammation induced by PM_2.5_ and HFD, including the activation of microglia in the hypothalamus. Rodents placed on HFD for up to 14 days displayed increased Iba1 in the medial basal hypothalamus, indicating microglia activation^15^. In the same way, our study shows an increase in Iba1 in ARH and PVH shortly after PM_2.5_ exposure (5 days). Besides, after 12 weeks of exposure to PM_2.5_, we observed an increase in the levels of mRNA of Ikbke, Tnf, and Tlr4 in the hypothalamus, which is comparable to long-term high-fat feeding, where the expression of Ikbke, Tnf, among others is elevated in the hypothalamus^15^. Despite increased inflammatory mediators after 12 weeks of exposure to PM_2.5_, we did not observe significant differences in Iba1 expression in the ARH, PVH, and VMH. This result may be due to a limited number of mice studied in each group. We had three mice in each group with heterogeneous results. Of note, an increase in the expression of any of the aforementioned genes impaired energy metabolism, as demonstrated^20,28,29^.

Our results are in accordance with previous studies that showed the pro-inflammatory effect of PM_2.5_ in the hypothalamus^16,17,42^. Several elegant studies demonstrated that central IKKβ, which is part of the canonical TLR4 pathway, was likely involved in the inflammatory signals in the hypothalamus of mice exposed to PM_2.5_ chronically. The inhibition of IKKβ by using different tools was able to reduce peripheral inflammation, contributing to decrease hepatic enzymes for gluconeogenesis and lipogenesis in mice chronically exposed to PM_2.5_. In another study, 16 weeks of PM_2.5_ exposure increased pulmonary, systemic and adipose tissue inflammation as well as induced endothelial dysfunction. The deletion of IKKβ by using nestin cre and IKKβ flox flox mice decreased these inflammatory responses^16,17,42^. So far, these mentioned studies have focused on effects of chronic exposure to PM_2.5_ on the peripheral disarrangements. Differently, our study focused on the acute and chronic effect of PM_2.5_ on energy metabolism changes involving leptin signaling and action. Besides, in our study, we investigated the non-canonical pathway of TLR4 involving IKKε in the hypothalamus and in parallel the canonical pathway seems to have a significant effect on the development of obesity.

The deletion, the knockdown or the pharmacological inhibition of TLR4 protects mice from obesity, inflammation, and leptin resistance^20–22,43^. Up to now, the major agonist of TLR4 is LPS, which has been found in the PM_2.5_^44^, representing a potential inflammatory pathway induced by air pollution. In this regard, it is possible to assume that elevated TLR4 expression in the hypothalamus may be due, at least in part, to exposure to PM_2.5_ and that a deletion of TLR4 before exposure to PM_2.5_ could protect mice from the development of leptin resistance and obesity. Leptin has a pivotal role in promoting inflammation^45^, and the presence of PM_2.5_ via inflammatory mediators induces the expression of leptin and LEPR^46^ forming a vicious cycle. Accordingly, healthy weight children chronically exposed to concentrations of fine particulate matter and ozone that exceed the recommendation (US EPA air quality standards) displayed higher leptin levels before they became obese^47^, reinforcing the potential inflammatory role of PM_2.5_ to regulate leptin secretion.

The non-canonical TLR4 signaling occurs in the endosomal fraction of the cell and involves adaptor proteins and the activation of IRF3, which in turn increases the expression of IKKε. Recently, it was shown that IKKε is increased in peripheral and hypothalamic tissues from obese mice^48^. The knockdown of Ikbke, specifically in the hypothalamus of obese mice decreased NFκB activation and improved leptin action^28^. Given the fact that IKKε plays a crucial role in the inflammatory process in the hypothalamus, it is hypothesized that IKKε signaling is required for PM-mediated hypothalamic inflammation and adiposity. Consistent with our hypothesis, we found that inhibition of IKKε signaling decreases adiposity in mice exposed to PM_2.5_. Furthermore, the knockdown of Ikbke gene improved leptin sensitivity, decreased food intake and increased energy expenditure, ameliorating insulin resistance. However, we cannot rule out the possibility that the improvement in both insulin and leptin resistance in mice exposed to PM_2.5_ and infused with si-IKKε was secondary due to the reduced adiposity in these mice.

The connections between the non-canonical TLR4 signaling and the suppressor of cytokine signaling 3 (SOCS3) expression is not yet well established, but there is evidence that this pathway can induce SOCS3 expression through the migration of NFκB to the nucleous of the cell^49^. As SOCS3 is a negative modulator of leptin signaling, a possible increase in SOCS3 could explain the reduction of leptin signaling in mice exposed to PM_2.5_.

It was observed a blunted anorexigenic response to leptin together with a reduction on STAT3 phosphorylation in the hypothalamus after 12 weeks of exposure to PM_2.5_, which is consistent with leptin resistance. An increase in STAT3 phosphorylation in response to leptin was observed in mice exposed to FA and PM_2.5_; however, the magnitude of STAT3 phosphorylation in response to leptin was attenuated by more than 50% compared to mice exposed to FA. Moreover, it is important to note that these mice received a standard chow diet, and a robust decrease in STAT3 phosphorylation in response to leptin is generally observed in rodents fed high-fat diet^50,51^. The leptin resistance induced by PM_2.5_ may contribute to the development of hyperphagia and reduced energy expenditure. Leptin is a major activator of POMC neurons and at the same time, an inhibitor of AgRP/NPY neurons^12,14^. Both NPY and AgRP have potent orexigenic effects^12,14^. However, despite been hyperphagic, mice exposed to PM_2.5_ did not display increased levels of AgRP/NPY gene in the hypothalamus. Instead, they exhibited reduced levels of POMC gene in the hypothalamus, which might contribute to increase food ingestion and to decrease energy expenditure.

In conclusion, our data demonstrate that short-term exposure to PM_2.5_ increases inflammation in the hypothalamus, similar to a HFD. Long-term exposure to PM_2.5_ is even worse, leading to leptin resistance, hyperphagia, and a decrease in EE due to inflammation in which TLR4 and Ikbke have a role. Thus, we suggest that air pollution affects energy homeostasis through the activation of hypothalamic inflammatory signaling and leptin resistance in mice.

## Methods

### Ethical approval

All methods and animal handling were performed following the National Institute of Health guidelines for the use of experimental animals with the approval of the Care of Animals and Ethical Committee for Animal Research of the State University of Campinas under the numbers CEUA 3872-1 and CIBio 4628-1/2017.

### Diet and Exposure of animals to FA or PM2.5

All mice received a standard rodent chow (3.39 kcal/g; Nuvilab CR-1, NUVITAL QUIMTIA, Brazil) and water *ad libitum*. The ambient particle concentrator is located at the University of Sao Paulo (USP), in Sao Paulo, Brazil and has been used by several studies^52–56^. The ambient particle concentrator has several kinds of impactors, which are capable to separate fine particles according to aerodynamic sizes and concentrate them from ambient air. To perform the exposures, mice were placed in chambers connected to the equipment. One chamber received PM_2.5_ and another chamber received FA due to additional high-efficiency particulate air filter. We estimated the adjusted exposure dose of PM_2.5_ used in our study based on EPA’s current methodology (https://www.epa.gov/node/81739/view). The adjusted exposure dose of PM_2.5_ used in our study was 600 μg/m^3^ for 1 h of exposure. Considering the current average levels of PM_2.5_ in São Paulo city, which is around 25 μg/m^3^ (levels recommended by the “World Health Organization, 2005 Air Quality Guidelines: Global Update 2005” (https://www.who.int/), our adjusted exposure dose of PM_2.5_ is equivalent to 24 h of exposure of Sao Paulo city dwellers. The composition of PM_2.5_ at this location has been characterized along the course of the experiments consisting of black carbons, polycyclic aromatic hydrocarbon, and metal trace elements such as Na, Al, Si, P, S, K, Ca, Ti, V, Fe, Nu, Cu, Zn, Pb^52–56^. Animals returned immediately to their home cages after each exposure. Male C57BL/6 J or WT mice, and TLR4-deficient mice (TLR4^-/-^) were obtained by the multidisciplinary center for biological research from University of Campinas, SP, Brazil. Mice were 6-10 weeks old at the beginning of exposures and were randomly assigned to each group. The animal facility had constant light/dark cycle (12 h/12 h), room temperature (22 °C), humidity, and a high-efficiency particulate air filter (HEPA).

### ICV cannulation and IKKε inhibition

At 11 weeks of exposure, anesthetized (ketamine and xylazine) mice underwent stereotaxic surgery (stereotaxic ultra-precise #963; KOPF, Tujunga, CA) to implant a micro-osmotic pump (ALZET 1007D - DURECT CORPORATION, Cupertino, CA, USA) filled with small interfering RNA (si-RNA) to knockdown Ikbke, and one-side stainless-steel guide cannula (26-gauge, PLASTICS ONE, Roanoke, VA) at the lateral ventricle (AP −0.5 mm; L −1.3 mm; DV −2.2 mm)^28^. The sequences of si-RNA, as well as the doses were validated in a previously study from our group^28,57,58^. After surgery, the animals remained exposed for five more days to complete the 12 weeks of exposure. The cannula position was tested with angiotensin II and the measurement of water intake was done as described elsewhere^28,57–59^.

### Metabolic parameters

Food intake was recorded in a single housed free-fed mouse. Free fed mice were placed in an indirect open circuit calorimeter (oxymax deluxe system; COLUMBUS INSTRUMENTS, Columbus, OH, USA) and after one day of acclimation, oxygen (O2) consumption, carbon dioxide (CO2), heat production and RER were measured. Body and fat mass, blood glucose or serum glucose, serum insulin and leptin levels were obtained from overnight fasted mice.

### Evaluation of insulin resistance using the HOMA-IR index

In order to estimate the insulin resistance, it was used the homeostasis model assessment (HOMA-IR) for insulin resistance = ([fasting glucose (mmol/L) x fasting insulin (µU/mL)]/ 22.5)^60^.

### Leptin sensitivity

To test leptin sensitivity in mice without cannula, saline was injected over 3 consecutive days (basal food intake) followed by 2 days of leptin injections (leptin response) (0.5 µg/g of body weight; #ab9750; ABCAM, Cambridge, UK) and more 3 days of saline again (recovered food intake). Both, saline and leptin were injected IP twice daily (at 08:00 and 16:00). Food intake was daily measured and the average of the first 3 days of saline injection was used to calculate the basal food intake for each group and was considered 100% of food ingested. Then, the measurement of food intake during leptin and the last 3 days of saline injections were expressed as % of basal food intake^61^. To test leptin sensitivity in mice previously cannulated, leptin (10 ηg/µL) or saline was injected ICV in overnight fasted mice. Then, cumulative food intake was measured for 4, 8, 12 and 24 h^28,57,58^.

### Tissue collection for protein analysis by immunoblotting

Overnight fasted mice received ICV injection (saline or leptin: 10 ηg), and fifteen minutes later, we collected the tissues or IP injection (saline or leptin: 2 µg/g of BW), and forty-five minutes later we collected the tissues. Before tissue collection, mice were euthanized by decapitation after an overdose of a mixture of ketamine hydrochloride (240 mg/kg) and xylazine hydrochloride (30 mg/kg) via IP injection. Tissues were snap-frozen immediately in N_2_ and stored at −80 °C for further analysis as described previously^28,57–59^. Phospho-STAT3 antibody (#9131; CELL SIGNALING TECHNOLOGY, MA, USA) and anti-beta actin antibody (#FNab00869; FINE TEST; Hubei, China) were used for IB. The Gel Doc System (BIO-RAD UNIVERSAL HOOD III, California, USA) captured the images, and their software performed the quantifications used in this paper.

### Tissue collection for mRNA and gene expression by qPCR analysis

Mice were euthanized by decapitation after an overdose of a mixture of ketamine hydrochloride (240 mg/kg) and xylazine hydrochloride (30 mg/kg) via IP injection. Then, BAT and the hypothalamus were quickly harvested, frozen in N2, and stored at −80 °C until RNA analysis. As previously described^57,58^ the RNeasy Mini Kit (#74106; QIAGEN INC, CA, USA) provides purification of RNA from dissected tissues, and NanoDrop 2000/2000c Spectrophotometer (THERMO FISHER SCIENTIFIC, Waltham, MA, USA) was used to quantify and assess purity of RNA. We employed the high Capacity cDNA Reverse Transcription Kit (#4368814), TaqMan and Quant Studio 6 Flex Real-Time PCR System (#4485694) and data assist software from APPLIED BIOSYSTEMS (CA, USA) to get the relative expression levels of the genes. Primer sequences will be available under request.

### Assays

To determine the concentration of serum insulin, leptin and corticosterone we used commercial ELISA kits (#EZRMI-13K and #EZML-82K from MILLIPORE; Billerica, MA, USA and #ab108821 from ABCAM, Cambridge, UK). Enzymatic colorimetric method was used to measure serum glucose levels.

### Immunofluorescence

Mice were anesthetized and perfused transcardially. The brains were sliced and processed as previously described^62^. The immunofluorescence staining was done in brain sections washed in 0.02 mol/L potassium PBS, pH 7.4 (KPBS) solution followed by 1 h incubation in 3% normal donkey serum. Then, brain sections were incubated overnight in anti-Iba1 antibody (1:1000; #ab5076 from ABCAM, Cambrige, UK). The sections were washed in KPBS, incubated for 90 min in AlexaFluor488-conjugated secondary antiserum (1:500; JACKSON IMMUNORESEARCH, PA, USA), and mounted on gelatin-coated slides, covered with Fluoromount G medium (E.M.S.) and analyzed under a microscope. To quantify the immunostaining, the integrated optical density was obtained of each area of interest using the ImageJ software (http://rsb.info.nih.gov/ij) and subtracted by the integrated optical density assessed in adjacent areas with low staining (background).

### Statistical analysis

GRAPHPAD PRISM SOFTWARE (San Diego, CA, USA) was used to calculate the mean, standard deviation (SD), and for statistical analyses and graphics production. All the results were expressed as means ± SD. Unpaired two-tailed Student’s t-test (two tailed) or ANOVA (one, two -way) with post hoc test (Bonferroni) were performed. All statistical tests were indicated in the legends. P < 0.05 was considered statistically significant.

## Supplementary information

Supplementary Information.

Supplementary Information.
